# Seed Longevity and Ageing: A Review on Physiological and Genetic Factors with an Emphasis on Hormonal Regulation

**DOI:** 10.3390/plants13010041

**Published:** 2023-12-21

**Authors:** Michela Pirredda, Iris Fañanás-Pueyo, Luis Oñate-Sánchez, Sara Mira

**Affiliations:** 1 Departamento de Biotecnología-Biología Vegetal, Escuela Técnica Superior de Ingeniería Agronómica, Alimentaria y de Biosistemas, Universidad Politécnica de Madrid, Av. Puerta de Hierro 2, 28040 Madrid, Spain; michela.pirredda@upm.es; 2 Centro de Biotecnología y Genómica de Plantas, Universidad Politécnica de Madrid-Instituto Nacional de Investigación y Tecnología Agraria y Alimentaria, Campus de Montegancedo, Pozuelo de Alarcón, 28223 Madrid, Spain; iris.fananas.pueyo@upm.es

**Keywords:** seed longevity, seed ageing, hormonal regulation

## Abstract

Upon storage, seeds inevitably age and lose their viability over time, which determines their longevity. Longevity correlates with successful seed germination and enhancing this trait is of fundamental importance for long-term seed storage (germplasm conservation) and crop improvement. Seed longevity is governed by a complex interplay between genetic factors and environmental conditions experienced during seed development and after-ripening that will shape seed physiology. Several factors have been associated with seed ageing such as oxidative stress responses, DNA repair enzymes, and composition of seed layers. Phytohormones, mainly abscisic acid, auxins, and gibberellins, have also emerged as prominent endogenous regulators of seed longevity, and their study has provided new regulators of longevity. Gaining a thorough understanding of how hormonal signalling genes and pathways are integrated with downstream mechanisms related to seed longevity is essential for formulating strategies aimed at preserving seed quality and viability. A relevant aspect related to research in seed longevity is the existence of significant differences between results depending on the seed equilibrium relative humidity conditions used to study seed ageing. Hence, this review delves into the genetic, environmental and experimental factors affecting seed ageing and longevity, with a particular focus on their hormonal regulation. We also provide gene network models underlying hormone signalling aimed to help visualize their integration into seed longevity and ageing. We believe that the format used to present the information bolsters its value as a resource to support seed longevity research for seed conservation and crop improvement.

## 1. Introduction

Loss of agrobiodiversity is a growing threat due to the abandonment of local varieties in favour of commercially driven, genetically uniform crops [1]. Now, a mere nine crops contribute to approximately 66% of global crop production [2]. Therefore, it is of paramount importance to preserve the genetic diversity of non-crop species for conservation purposes, but also as potential reservoirs of useful traits that could be reintroduced into elite cultivars. The establishment of germplasm banks, dedicated to storing seeds of various plant species and crop varieties, has become instrumental in safeguarding valuable genetic resources. The inevitable decline in seed viability with age is a critical factor determining their long-term storability, and thus, improving this characteristic is paramount for safeguarding wild species and crops’ genetic diversity and yield.

**Figure 1:**
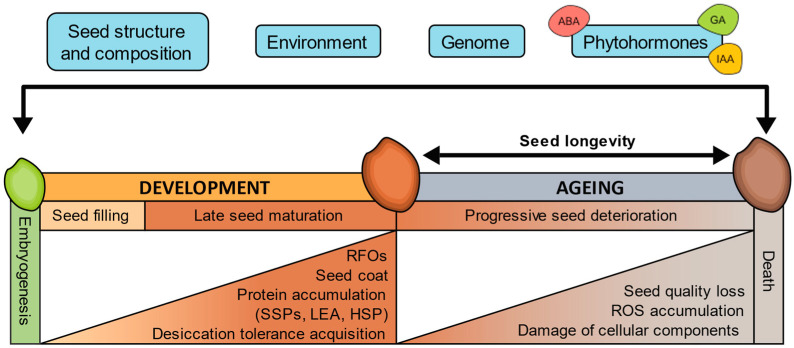
Factors affecting seed longevity and processes associated with seed development and ageing. ABA, abscisic acid; HSP, heat shock protein; IAA, auxins; GA, gibberellins; LEA, late embryogenesis abundant protein; RFOs, raffinose family oligosaccharides; ROS, reactive oxygen species; SSPs, seed storage proteins.

The period during which seeds maintain their viability determines longevity, which is significantly influenced by environmental conditions experienced during storage, including temperature, equilibrium relative humidity (RH), and oxygen pressure (Figure 1) [3,4,5,6,7,8]. Seeds can be classified into three primary categories based on their behaviour during storage: recalcitrant, intermediate, and orthodox [9,10]. Recalcitrant and intermediate seeds are extremely sensitive to desiccation and chilling, conditions routinely used for ex situ conservation of germplasm, thus making their storage problematic. Recalcitrant seeds have reduced longevity and are typically found in woody species from tropical or subtropical habitats [11,12]. Intermediate seeds, comprising around 10–15% of angiosperms worldwide, can tolerate higher levels of dehydration than recalcitrant seeds, but are not as resistant as orthodox seeds [10,13,14,15].

Orthodox seeds, which include most crops, are characterized by their ability to tolerate desiccation to moisture contents of 10% on a fresh weight basis or lower, and endure subfreezing temperatures, typically as cold as −20 °C [16]. Dry orthodox mature seeds reach a state characterized by extreme cellular viscosity, called a glassy or vitrified state, during which their cellular activities and metabolism are greatly slowed down, including oxidation [8,17,18,19,20,21]. This mechanism represents an important protection against ageing, allowing orthodox seeds to survive for long periods with a vitrified cytoplasm [22,23,24]. However, predicting seed longevity is challenging due to considerable variations among species and storage conditions [25,26,27]. Also, seeds subjected to the same storage conditions can lose viability at vastly different rates due to variations in their physiological and genetic characteristics, with hormone signalling emerging as prominent regulators of seed longevity [3,25,26,28]. Understanding the factors that contribute to the longevity of seeds and how they interact can provide valuable insights for seed conservation and agricultural practices.

This review first explores the mechanisms of seed ageing in orthodox species and how they are influenced by seed storage conditions. The second part is focused on phytohormone signalling pathways that regulate seed ageing and provide a summary of genes involved in hormonal signalling and seed longevity. Thus, this review aims to shed light on the intricate mechanisms governing seed longevity and provide valuable insights for future research, leading to the improvement of this trait. Unless otherwise indicated, genes mentioned throughout this review are from *Arabidopsis thaliana*, and At in front of the gene names has been omitted for simplicity.

## 2. Seed Ageing Mechanisms

Seed ageing is a natural and irreversible process that leads to a progressive deterioration of seed quality. Initially, it manifests as a delay in the germination speed, followed by a gradual loss of viability evidenced by an increase in the percentage of seeds unable to germinate, and eventually culminates in the death of all seeds in the lot. This degenerative process occurs over time, even under optimal storage conditions [29]. Numerous physiological and biochemical changes have been linked to seed ageing, including the accumulation of reactive oxygen species (ROS), lipid peroxidation, loss of membrane phospholipids, reduced activity of antioxidant enzymes, and volatile production [30,31,32,33,34]. Additionally, ageing is associated with changes in transcript levels, impaired protein synthesis and post-translational modifications such as protein inactivation by carbonylation, hydrolysis, or alteration in enzyme activity [35,36,37]. So, ageing appears to result from a gradual decline in the enzymatic activities responsible for repairing damage, ultimately leading to the accumulation of unrepaired oxidative damage.

Recent genome-wide and reverse genetics studies on *Arabidopsis thaliana* highlighted the important role of oxidative stress on seed ageing, identifying genes involved in ROS metabolism and detoxification as related to seed longevity [38]. ROS originating from the partial reduction of oxygen can cause oxidative damage when their levels exceed the homeostatic capacity of the seed antioxidant system, particularly under stress conditions [39,40]. These highly reactive chemicals interact with cellular biomolecules, leading to severe oxidative damage in proteins, nucleic acids, and lipids [41]. The production of ROS in seeds depends on their metabolic and physiological state [42]. In hydrated seeds, the primary source of ROS is the mitochondrial respiratory chain, especially during germination when respiratory activity increases [43,44]. When seeds are in a dry, glassy state, such as during storage in dry conditions, the enzyme activities producing and scavenging ROS are extremely reduced. However, even in this state, ROS continue to accumulate due to non-enzymatic reactions, such as lipid peroxidation [45]. Although ROS accumulation is linked to seed ageing processes, cells are equipped with detoxifying enzymes and antioxidant compounds like glutathione to balance the effects of ROS and ensure seed survival [43]. When stored seeds are imbibed and set off to germinate, the accumulation of ROS is counteracted by the antioxidant system, and it has been recently identified that concentrations of glutathione before ageing provided a predictive indicator of seed longevity across a range of Arabidopsis wild-type accessions [46]. However, as seeds age, ROS-processing enzymes are themselves affected by oxidative damage, and the resulting decline in antioxidant enzyme activity compromises ROS detoxification, a situation that negatively affects seed longevity [47,48,49,50,51].

Among the various oxidative processes contributing to seed ageing, lipid peroxidation plays a crucial role. This process involves the oxidative degradation of lipids, particularly from the cell membrane, leading to cell damage and negatively impacting seed viability [42,52]. Studies based on the determination of malondialdehyde content have shown that lipid peroxidation is linked to seed ageing in different species [53,54]. However, contrasting results not finding a correlation between this compound and ageing have also been reported [47,49]. Since lipid peroxidation alters the membrane’s permeability, producing an increase in solute leaching, electrical conductivity measurements have also been used as a parameter to quantitate seed ageing in various species, specially within the Brassicaceae family [49,55,56,57,58,59,60]. Similarly, by measuring lipid peroxidation, some studies have not shown a direct correlation between electrical conductivity and seed ageing [61]. Since the rate of lipid peroxidation depends on the equilibrium RH and the seed lipid content, differences between species and experimental storage conditions may underly the contradictory results [32,33,62].

DNA and RNA integrity can also be affected during storage, especially due to oxidative stress. ROS are known to induce genotoxic effects in seeds, compromising the stability of DNA [34,63]. As seeds age, point mutations accumulate, and structural damage such as single and double-strand breaks could lead to DNA fragmentation [52,63,64,65,66,67,68]. Seeds may still be capable of germination despite genotoxic stress, but they must overcome damages in DNA to prevent mutations from being passed on to future generations [69,70,71]. To achieve this, DNA repair mechanisms are activated in the embryo during early germination stages, and DNA repair enzymes have been found to play a key role in seed longevity [71,72,73]. Recent studies highlight the crucial role of RNA metabolism in regulating seed longevity. Degradation processes of RNAs have been linked to seed ageing in various species [74,75,76]. In soybean seeds, it has been observed that lower viability is associated with RNA fragmentation during the ageing process [75]. This damage seems to occur randomly throughout the seed transcriptome, suggesting that RNA fragmentation could serve as a consistent molecular marker for tracking the progression of ageing in dry seeds. Moreover, the regulation of mRNA storage and processing has been identified as a significant factor in seed longevity. Specifically, factors related to RNA spliceosome subunits MOS4 or MAC3A/MAC3B have been highlighted and allow for the differentiation of accessions with high and low seed longevity [46].

The role of DNA methylation as a potential indicator of seed ageing and viability loss during seed storage has garnered attention, although investigations into epigenetic changes during this process remain limited [77]. Studies have revealed that drying has an impact on the global level of DNA methylation in seeds of certain tree species [78,79,80]. Also, seed ageing increased epigenetic instability in both seeds and seedlings in several species [81,82]. In mint seeds, methylation changes after storage were detected at a low rate, but changes increased in seedlings produced from aged seeds [82]. Supporting these results, rye seeds showed epigenetic changes in stored seeds and seedlings [81]. Understanding the impact of ageing on DNA and RNA integrity and their epigenetic marks, as well as the mechanisms involved in repairing and overcoming such damages, is critical for ensuring seed viability and preserving genetic stability in plants.

## 3. Seed Storage Conditions: Key Role of Water Content

Diverse experimental storage approaches have been used on seed ageing research, which led to variations in outcomes between different studies, making comparisons difficult. Equilibrium RH, temperature, and atmosphere are the main factors determining seed longevity during storage, and have been the focus of numerous studies since the 1990s [3,6,7,52,83,84]. The water content of seeds governs the biophysical and biochemical state of cells during storage and is recognized as a critical factor dictating the process of seed ageing [7,8,17,19,33,85,86,87,88,89,90]. Consequently, seed ageing mechanisms show substantial differences between storage under high or low equilibrium RH [32,33]. However, defining specific boundaries for what constitutes high or low humidity proves challenging, and terms like “accelerated ageing”, “controlled deterioration”, or “natural ageing” are rather subjective and often used imprecisely [91,92].

Another crucial aspect that contributes to the divergent outcomes in seed longevity studies is the choice of containers for storage. The importance of controlling humidity and storage atmospheres in deterioration experiments necessitates the use of hermetic containers. However, many lab containers fall short of providing sufficient hermeticity for extended periods of high humidity control [93]. Moreover, some experiments rely on hermetic storage using sealed containers, such aluminium foil bags or glass jars, while others employ open containers inside an incubator. The type of storage, whether hermetic or open storage, has been identified as a critical factor influencing seed longevity performance, likely due to variations in oxygen exposure, which is limited in hermetic conditions [84]. Also, studies have explored the impact of different gaseous environments, including air, vacuum, CO_2_, and N_2_, on seed viability during storage [94,95,96]. Oxygen presence in the storage environment has been identified as particularly harmful, leading to faster viability loss [4,97]. These findings indicate the importance of the storage containers and gas atmosphere as a factor affecting seed longevity.

Extrapolating findings obtained at one experimental condition to different storage settings further complicates matters. The rate and type of biochemical reactions might not be comparable when ageing is assayed at 100% or 75% equilibrium RH since, for example, the onset of respiration is at 90% RH (approximately water content of 0.25 g H_2_O g^−1^ dry weight) for a wide range of species [15,89]. Although many species age faster both at high RH and also in dry storage [25], some species exhibit unexpected faster ageing in the latter condition [62]. In Arabidopsis, relative longevity was compared among natural ecotypes, and similar results were found for two storage conditions below 75% RH, but not for storage above 75% RH [38,46]. In the case of studies with mutants, some of them rendered similar results when assayed under wet and dry conditions, while for heat shock protein mutants, the results were greatly influenced by storage conditions [38,98,99,100].

These observations highlight the significant disparity in ageing mechanisms depending on the storage conditions, thus limiting the predictive capacity of storage experiments for seed longevity. To address this issue, Hay et al. [91] proposed the use of ageing conditions that align with the intended future applications of the research results, and if possible, implement standardized conditions to facilitate comparison across studies. Following these recommendations, Zinsmeister et al. [28] coined three terms to classify seed ageing conditions based on association with a range of experimental values, instead of fixed values, for equilibrium relative humidity and temperature: (1) “Wet ageing”, which encompassed the accelerated ageing and controlled deterioration tests, storing seeds at high RH (>80%), and a wide range of temperatures (4–45 °C); (2) “Intermediate ageing”, which would allow the study of seed longevity under dry conditions within a reasonable timeframe by storing seeds at 75% RH and 30–35 °C; and (3) “Dry ageing”, representative of both natural ageing and seed bank conditions for long-term storage, storing seeds at low RH (<70%), and temperatures from 25 to −20 °C. In this latter case, given the wide range of temperatures associated to dry ageing, its selection is not trivial since it may introduce considerable variability in the experimentation. This classification will be used in this manuscript to categorize the storage conditions of different studies and allow comparisons.

## 4. Other Factors Affecting Seed Longevity

Seed longevity is a multifaceted trait influenced by the genome and seed structural composition, which are themselves subjected to environmental cues [25,26,101,102,103,104,105,106,107]. Interestingly, seed longevity exhibits similarities within some particular taxonomic families or genus, like the Apiaceae, being their seeds consistently short lived [25,26]. On the contrary, this trait can vary significantly between species within the same family [25,26], between accessions of the same cultivar [108,109,110], or even between seeds from the same plant [111,112]. It is of high interest to investigate whether those taxonomic-associated patterns of seed longevity are related to similarities or differences in seed structure and composition, including traits like lipid thermal behaviour, volatile production, and seed coat structure [25,32,33,55,113].

Longevity is progressively acquired during the final stages of orthodox seed development and maturation. These stages are associated with seed filling, acquisition of desiccation tolerance, loss of chlorophyll, and accumulation of the raffinose family oligosaccharides (RFOs) and seed storage proteins (SSPs) [114,115,116,117,118]. These processes have been related to seed longevity and are regulated by genetic programs that integrate hormonal and metabolic pathways mainly controlled by LAFL (i.e., LEC1, ABI3, FUS3, and LEC2) transcriptional regulators [115,119]. The LAFL regulatory network includes the family of B3 domain transcription factors ABI3 (ABSCISIC ACID INSENSITIVE3), FUS3 (FUSCA3), and LEC2 (LEAFY COTYLEDON2); and LEC1 (LEAFY COTYLEDON1), a member of the NFYB protein family [120]. Arabidopsis *abi3* and *lec1* mutants, in addition to showing a decrease in SSPs, RFOs, and chlorophyll degradation, also have reduced longevity [119]. Along with genetic regulation, environmental conditions during seed development have the potential to exert a profound influence on seed longevity [103,121]. Studies have demonstrated the significance of environmental parameters in shaping seed longevity traits, with light intensity and temperature during seed development showing positive correlations with longevity [122]. Consequently, species originating from cool, temperate climates tend to produce short-lived seeds, while those from warm, arid environments yield seeds with extended lifespans [25,26,102,121,123].

Seed coat structure plays an important role in seed longevity since it serves as a protective layer that shields the embryo from external abiotic and biotic factors [38,124]. Seeds of Arabidopsis *transparent testa* mutants lack proper regulation of testa composition and show reduced longevity [125]. Also, the identification of several seed-coat-related genes in regulating longevity supports its protecting role during storage [38,126]. Cell wall structure is crucial for seed viability maintenance, and recent studies suggest that genes involved in callose degradation can be related to both longevity and dormancy [127].

Seed composition, particularly lipid content, has been associated with variations in longevity among species. While the notion that high lipid content leads to lower longevity has been widespread [56], recent analyses have shown that the lipid composition plays a more significant role due to differences in susceptibility to oxidation or thermal behaviour [17,52,113,128]. For example, the energy associated with lipid melting has been used as a descriptor of viability loss [129]. Sugars and proteins also play a crucial role in seed longevity by stabilizing the glassy cytoplasm [20]. Non-reducing sugars, mainly sucrose and RFOs, heat shock proteins (HSPs), and late embryogenesis abundant (LEA) proteins, are synthesized during seed development and contribute to seed desiccation tolerance and longevity, likely preventing aggregation in the glassy-state and assisting in protein folding [116,118,130,131,132,133,134,135]. SSPs accumulate during seed filling and are major targets for oxidation and carbonylation [136]. This makes them an important part of the seed’s defence against oxidative damage, protecting other proteins from oxidation during storage. In Arabidopsis seeds, cruciferins and napins are the two main types of SSPs. Studies involving comparative proteomics and knockout mutants have identified these proteins, cruciferins in particular, as crucial for seed longevity [137,138].

In summary, it is evident that seed structure and chemical composition have a pivotal role as key endogenous factors influencing seed longevity. Therefore, gaining a better understanding of the factors that regulate these processes offers a new approach to bolster seed longevity.

## 5. Hormone-Mediated Regulation of Seed Longevity

In recent years, research on hormonal signalling pathways related to longevity has sparked increased interest. In particular, signalling mediated by abscisic acid (ABA), auxins, and gibberellins (GAs) have emerged as key players affecting this trait given their profound influence on seed development, composition, and structure. This section aims to explore the impact of hormonal regulation and signalling pathways on seed longevity and provide detailed information on the specific experimental conditions to better evaluate commonalities and differences between different plant species. Three different seed storage conditions have been used in the publications reviewed in this section that can be classified according to Zinsmeister et al. [28].

### 5.1. Abscisic Acid

ABA plays a significant role in enabling plants to resist various stresses, particularly those caused by abiotic factors, and is antagonized mainly by GAs [139,140,141,142]. ABA is known to repress seed germination, but it is required for crucial processes occurring during seed development, such as accumulation of seed-storage compounds, establishment of seed dormancy, and acquisition of desiccation tolerance [143,144,145,146]. Consequently, ABA plays a key role in seed longevity acquisition by regulating gene expression associated with seed dehydration tolerance and synthesis of RFOs, HSPs, and LEA proteins [135,140,147,148].

The ABA-signalling pathway relies on three key early components. Binding of ABA by the PYR/PYL/RCAR (pyrabactin resistance/pyrabactin resistance-like/regulatory component of ABA receptor) proteins triggers deactivation of the PP2Cs (type 2C Ser/Thr protein phosphatases). The absence of PP2Cs allows activation of the SnRK2 (Snf1-related protein kinase class 2) kinase that phosphorylate downstream signalling components [149,150]. The *LAFL* genes are master regulators controlling gene expression programs intimately linked to ABA signalling and essential for seed maturation, accumulation of storage compounds, and seed longevity. Their core function is well conserved among the spermatophytes, and although LAFL mutants alter seed longevity, they also compromise several other aspects related to their wide regulatory roles (i.e., seed filling, acquisition of desiccation tolerance, loss of chlorophyll, and accumulation of RFOs and SSPs). Several genes controlled by ABA/LAFL-signalling have been found to play a role in seed longevity. ABI3 is a transcription factor that positively regulates the ABA-signalling pathway, and *ABI3* mutants show ABA insensitivity and reduced accumulation of seed storage proteins and seed longevity [119,143,151,152,153]. Clerkx et al. [154] reported that mutations such as *abi3* or *aba1* (*abscisic acid deficient1*; ABA biosynthetic mutant) led to reduced seed longevity under different ageing conditions. Tesnier et al. [155] and Sugliani et al. [119] also demonstrated poor longevity in ABA-related mutants, emphasizing the crucial role of *ABI3* and *LEC1* in maintaining seed viability in Arabidopsis during storage. Interestingly, co-expression network studies on the legume *Medicago truncatula* seeds have indicated that seed longevity is regulated by both MtABI3-dependent and independent pathways [115]. Downstream ABI3, the Arabidopsis transcription factor HSFA9 (HEAT SHOCK FACTOR A9) and its homologues in *M. truncatula* and soybean have also been linked to seed longevity [114,116,156,157,158,159]. In fact, heat shock transcription factors (HSFs) such as HSFA9 promote the expression of small heat shock protein genes (sHSP), contributing to a genetic program that regulates embryonic desiccation tolerance and seed longevity in different species [99,115,158,160,161,162,163]. In Arabidopsis, HSFA9 promotes the expression of HSPs and tonoplast intrinsic proteins (TSPs; aquaporins) mediating resistance against seed deterioration during storage [156,161,162]. Mutations in two Arabidopsis aquaporins localized in the seed protein storage vacuole membrane, *tip3-1/tip3-2*, exhibited reduced seed longevity after storage, and ABI3 was found to bind the *TIP3* promoters and required for enhanced *TIP3* mRNA and protein levels [152]. Moreover, *hsfa9* mutant seeds of Arabidopsis, *M. truncatula,* and tobacco lost viability faster than wild-type seeds when stored at wet ageing and exhibited deregulation of ABA biosynthesis-related genes [99,158,161]. However, longevity of *hsfa9* seeds was comparable to that of the wild-type seeds at intermediate ageing conditions, suggesting that *HSFA9* plays a role in thermotolerance rather than in dry seed storage [99,158,161]. In summary, ABI3 and HSFA9 constitute an important ABA-related regulatory module for seed longevity that seems to be conserved in several plant species.

Seed longevity is established during the late maturation stage, coinciding with the process of seed degreening. It is long known that chlorophyll retention in mature seeds can be an undesirable trait, being related to low seed storability [164,165]. Chlorophyll degradation during seed maturation is also regulated by the ABA signalling pathway, and *abi3* mutants are not able to degrade chlorophyll during maturation and remain green even after desiccation [143,151,166,167]. In this regard, the Arabidopsis *nyc1* mutant (*Non-yellow coloring1*), encoding a chlorophyll b reductase isoform, showed increased seed chlorophyll content and exhibited a rapid decline in viability during storage at room temperature compared to wild-type seeds. Not surprisingly, *NYC1* expression was repressed in the *abi3* mutant, suggesting that ABA induces chlorophyll degradation during seed development to retain storability [166]. ABI4 is an AP2 domain-containing transcription factor and another core component of the ABA signalling pathway during seed development [168,169]. A role for *ABI4* in longevity of *M. truncatula* and *Solanum lycopersicum* (tomato) seeds had previously been hinted at by gene co-expression analyses [116,148]. Indeed, Arabidopsis and *M. truncatula abi4* mutants have reduced longevity compared to wild-type when stored at intermediate ageing conditions, and also showed elevated chlorophyll levels and reduced dormancy [170].

Additionally, another transcription factor of the ABA signalling pathway, ABI5, has been shown to be an important regulator of longevity in legume seeds [171]. In *M. truncatula*, MtABI5 is a positive regulator of seed longevity, dormancy, and storage proteins, and also act in the regulatory network of degreening during late seed maturation. *Abi5* mutant seeds of pea and *M. truncatula* retained some chlorophyll and exhibited decreased longevity [171]. Moreover, the analysis of *Mtabi5 Mtabi4* double mutants showed synergistic effects on chlorophyll retention and longevity, suggesting that they act via parallel pathways [170]. Consequently, ABI4 is hypothesized to coordinate the dismantling of chloroplasts during seed maturation and the subsequent attainment of seed longevity, acting in synergy with ABI5. In Arabidopsis, ABI5 controls the genes related to chlorophyll metabolism, similar to legumes. However, when ABI5 is not fully functional in seeds (*abi5* mutants), seeds do not display green coloration or shortened longevity [171]. This might be attributed to the potential regulation of ABI5 by DOG1 (DELAY OF GERMINATION 1), ABI3, and a group of redundant bZIP (basic leucine zipper) transcription factors homologous to ABI5 (homologous ABF genes) [167,171]. All these regulatory proteins play a vital role in controlling the process of acquiring seed longevity.

As previously mentioned, dormancy is another process strongly related to longevity. The Arabidopsis *DOG1* gene plays a crucial role in the establishment of seed dormancy during seed development [172]. Seeds of the *dog1* loss-of-function mutants show reduced longevity under dry conditions [173]. Through transcriptomics and metabolomics approaches, Dekkers et al. [167] found that the DOG1 function is also required at later stages of seed development for seed longevity acquisition by promoting the expression of numerous genes, such as *HSPs* and *LEAs*, partly through positive control of *ABI5* expression and in conjunction with *ABI3* signalling. However, a negative correlation between seed longevity and seed dormancy was reported by analyses with inbred line populations [174]. The results revealed five loci (*GAAS)* that are collocated with *DOG* seed dormancy loci. Detailed analysis on the collocating *GAAS5* and *DOG1* quantitative trait loci revealed that the *DOG1*-Cape Verde Islands allele both reduces seed longevity and increases seed dormancy. These results suggest the existence of separate mechanisms for seed dormancy and longevity that may vary between accessions as a result of adaptation to its natural environment. Interestingly, we have just found that a DOG1 downstream target gene negatively feedback on *DOG1* expression and dormancy, and enhances germination without altering seed longevity [175].

Several studies indicate that the role of ABA as a positive regulator of seed dormancy may not be indisputable. A negative regulator of ABA signalling was isolated as a dominant Arabidopsis mutant by screening an activation-tagging mutant collection [98]. This mutant showed increased seed longevity caused by a higher expression of *RSL1* (Ring finger of Seed Longevity1), a gene encoding a RING-type zinc finger putative ubiquitin ligase. Interestingly, RSL1 reduced ABA sensitivity and altered regulation of ABA-responsive gene expression through the interaction with receptors PYR1 and PYL4 [176,177]. Although the seed’s longevity phenotype was unaffected by the specific ageing conditions used during the screening process (dry or wet ageing), reduced seed longevity for the *rsl1* loss-of-function was only observed under wet ageing conditions [98], therefore highlighting the relevance of experimental conditions for assessing seed ageing. More evidence linking ABA-signalling with reduced longevity came from a study with Arabidopsis *DREB2B* and cotton orthologous *GhDREB2B* (Dehydration-Responsive Element-Binding Protein 2B) [178]. DREB2B belongs to a subgroup of the DREB transcription factor family and was found to function as a negative regulator of seed longevity after wet ageing. This function seemed to be under the control of an ABA-mediated pathway, since the *abi3-16* mutant allele enhanced seed longevity of the *dreb2b* mutant, suggesting that ABI3/DREB2B negatively regulates seed longevity in both Arabidopsis and cotton species [178].

Regardless of seed longevity being under control of ABI3 (master regulator), bioinformatic and comparative analyses of expression during seed maturation data from *M. truncatula* and Arabidopsis suggested that at least half of the longevity-regulated genes are controlled by ABI3-independent pathway/s [115]. In support of this finding, Righetti et al. [115] identified two transcription factors (*NFXL1* and *WRKY3*) whose mutants produced seeds with reduced permeability and longevity but no alterations in ABA sensitivity or *ABI3*-related traits (dry weight, chlorophyll degradation, accumulation of non-reducing sugar contents) [115]. In addition, these two transcription factors do not seem to be targets of ABI3, LEC1, and LEC2 [179,180,181]. Also, a transcriptomic study [148] characterizing the acquisition of longevity during seed maturation revealed 14 genes whose expression strongly correlated with longevity. Among them, Bizouerne et al. [148] found orthologs of known Arabidopsis regulators of ABA signalling pathways, such as PYL5, an ABA receptor, and RAV1 (related to ABI3/VIP1), a B3 transcription factor repressing the expression of *ABI3*, *ABI4*, and *ABI5*. Regarding the biochemical ageing mechanisms, seed deterioration is related to the accumulation of DNA damage, and recent findings point to the WHIRLY (WHY) family of DNA-binding proteins as important players in the maintenance of nuclear and organellar genomes. Longevity of *why1* and *why3* Arabidopsis mutants was lower than the wild type after wet ageing, and the double mutant *why1why3* was highly sensitive to ageing [182]. In addition, seeds of the *why1* mutants are insensitive to the phytohormones ABA and salicylic acid during germination [183].

**Figure 2:**
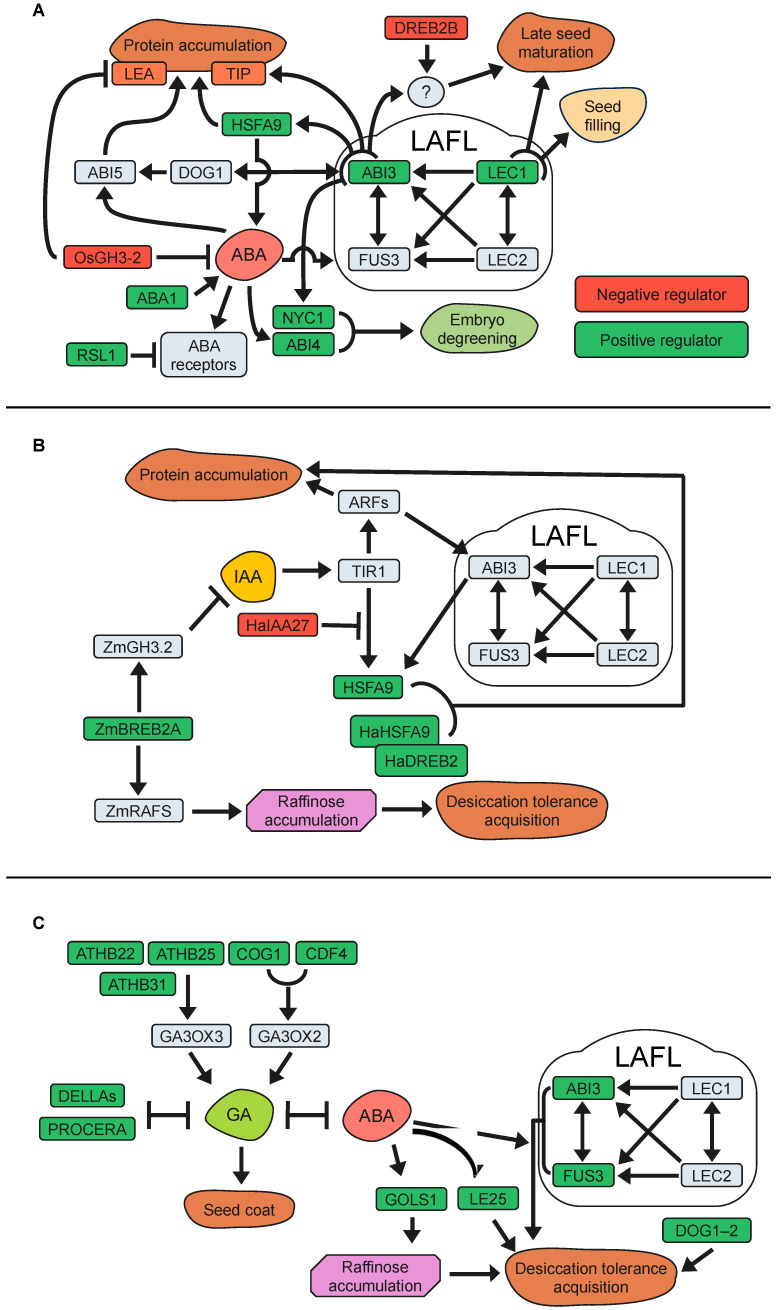
Phytohormone-related genes with effects on seed longevity. (**A**) Graphic representation of genes related to abscisic acid (ABA); (**B**) genes related to auxins (IAA) and (**C**) genes related to gibberellins (GA) signalling pathway.

In summary, seed longevity and the influence of important ABA-related genes have been studied in one or various plant species (Table 1, Figure 2A). Although most findings highlight the significance of ABA-related mechanisms as positive regulators of seed longevity across different plant species and storage conditions, some results deviate from this common thread, thus underscoring the complexity of this regulation.

### 5.2. Auxins

Auxins are plant hormones whose primary function involves cell division, differentiation, fruit development, root formation, apical dominance, and leaf fall (abscission) [184]. Additionally, auxins are associated with critical aspects of seed characteristics, including size, weight, dormancy, and seed coat development [146,185,186]. One of the most significant natural hormones among auxins is the ß-indoleacetic acid, abbreviated as IAA (the main auxin in plants). The core components of the auxin-signalling machinery belong to three protein families: the F-box TIR1/AFB auxin co-receptors (TRANSPORT INHIBITOR RESPONSE 1/AUXIN SIGNALING F-BOX PROTEIN), the Aux/IAA transcriptional repressors (Auxin/INDOLE-3-ACETIC ACID), and the ARF transcription factors (AUXIN RESPONSE FACTOR). Auxin promotes an interaction between TIR1/AFB and Aux/IAA proteins, resulting in degradation of the Aux/IAAs and the release of ARF repression [187].

Using auxin biosensors, Pellizaro et al. [146] showed changes in auxin distribution and signalling, going from highly localized to widely spread throughout the embryo during the final stages of seed maturation. Also, using auxin single mutants, they found that seed longevity was affected in a dose-response manner depending on auxin levels. Moreover, the positive effect of auxins on seed longevity required ABI3 function, while the *ABI3* gene and genes with promoters enriched in ARF-binding sequences were induced by auxin in developing embryos, pointing to an epistatic relationship between auxin and ABA in the control of seed longevity. This relationship was strengthened by employing a gene co-expression network analysis in *M. truncatula* and Arabidopsis. These investigations identified a high number of auxin- and HSP-related genes, suggesting the potential connection between seed longevity regulation and increased auxin levels during seed maturation, possibly through interactions with *HSFA9*, an ABI3-regulated gene [115]. In sunflowers (*Helianthus annuus*), a study examined the connection between auxins, seed longevity, and ABA by investigating the interactors of the HaHSFA9 protein [156]. The research revealed that the Aux/IAA protein HaIAA27 opposes the transcriptional activation mediated by HaHSFA9. This repression can be reversed by treating sunflower embryos with auxin. This finding suggests that, in addition to ARFs, the Aux/IAA-signalling pathway is recruiting an HSF protein induced by ABA to regulate seed longevity.

Regulation of IAA biosynthesis might have a potential role on seed longevity, as suggested by the positive association of an IAA-biosynthetic gene (*CYP79B2*) with seed longevity in Arabidopsis [146]. Moreover, IAAs can be inactivated by conjugation with amino acids catalysed by auxin-amido synthetases, and *GH3-2* (GRETCHEN HAGEN3-2), a gene encoding one of these auxin-amido synthetases, has been negatively correlated with seed longevity [188,189,190]. In rice (*Oryza sativa*), functional analyses indicated that the *OsGH3-*2 encoded enzyme is involved in auxin inactivation, and its gene expression levels have a positive correlation with reduced ABA levels, ABA-related gene expression, and reduced seed longevity [189]. Additional research has shown that the overexpression of specific DREB family members, such as *DREB1A* (*CBF3*) in Arabidopsis and *ZmDREB4.1* in tobacco, can decrease IAA accumulation in plant tissues [191,192]. Interestingly, experiments on maize (*Zea mays*) indicate that *zmdreb2a* mutants have reduced expression of *ZmGH3.2,* but increased IAA levels in their embryos and reduced seed longevity [190]. These apparently contradictory results could be explained by the simultaneous reduction of raffinose content in the *zmdreb2a* embryo, attributable to the lack of direct DREB2A transcriptional stimulation of ZmRAFS (*RAFFINOSE SYNTHASES*). These results suggest that the transcription factor ZmDREB2A regulates the expression of *ZmRAFS* and therefore the accumulation of RFOs, which can influence seed longevity. However, the molecular mechanisms by which DREBs regulate IAA levels in plant cells are not yet fully understood, and data available so far emphasize the implication of multiple molecular interactions. The most important IAA-related genes and their relationship with seed longevity have been compiled in Table 1 and Figure 2B.

### 5.3. Gibberellins

GAs are compounds that belong to the tetracyclic diterpenoid family, some of which are bioactive growth regulators in plants, fungi, and bacteria [193]. In plants, GAs control diverse aspects of growth and development, and they have also been associated with agronomic traits such as seed size, weight, dormancy, and germination [194,195,196]. Genetic screenings have identified three main components in GA perception and early GA signalling: the GID1 GA receptors (GIBBERELLIN INSENSITIVE DWARF1) and the SLY1 F-box protein (SLEEPY1), which are positive regulators of GA signalling, and the DELLA proteins, which play a negative role in GA-regulated expression and are targeted for degradation in the presence of GA [197]. DELLA proteins belong to the GRAS (GIBBERELLIC ACID INSENSITIVE REPRESSOR OF *ga1-3* SCARECROW) family of transcription factors, acting as pivotal repressors in the GA signalling pathway [198]. In Arabidopsis, five DELLA proteins have been described (RGA: REPRESSOR OF GA1-3; GAI: GA-INSENSITIVE; RGL1: RGA-LIKE 1; RGL2: RGA-LIKE 2; and RGL3: RGA-LIKE 3), while only one has been identified in tomato (PRO: PROCERA) and cereals [199,200,201].

The positive role of GAs in seed longevity was well highlighted in Arabidopsis, demonstrating an increase in viability after wet storage of seeds originating from GA_3_-treated plants or a DELLA mutant with constitutive GA-signalling [98]. The authors also found that over-expression of the transcription factor *ATHB25* (*ARABIDOPSIS THALIANA HOMEOBOX25*) increases GA levels by enhancing the expression of *GA3ox2* (*GIBBERELLIC ACID3-OXIDASE2*), encoding a GA biosynthetic enzyme. Previous observations suggested that GAs in the outer integument would be required for the normal formation of the Arabidopsis seed coat [202]. Interestingly, it was observed that ATHB25 has an impact on seed coat structure and permeability by regulating lipid accumulation in the testa, a function also present in tomato and wheat [38,98]. In addition, Bueso et al. [203] reported that the overexpression of the *COG1* and *CDF4* genes, two DOF transcription factors, improves seed longevity. In particular, *COG1* is involved in seed coat development and increases the level of GA_1_ in siliques, probably due to the up-regulation of the *GA3ox3* biosynthetic gene. This evidence has clearly connected GA-mediated seed coat development, changes in testa structure, and changes in composition with seed longevity.

The role of DELLA proteins and their involvement in seed dormancy and longevity has also been studied among different plant species. In these cases, a negative relationship between GA-signalling and longevity was found, suggesting that the positive effects mentioned above could result from a DELLA-independent mechanism. A study in tomato identified that mutant seeds with no DELLA activity (*procera* mutant) showed reduced longevity during dry and intermediate storage, pointing to the negative involvement of the GA-signalling pathway on seed longevity [148,201]. In addition, the *procera* mutant also showed a reduction in the expression of desiccation-tolerance-related genes known to be upregulated by ABA, thus underscoring that the antagonistic balance between ABA and GAs is also involved in the regulation of seed longevity [201]. PROCERA has also been shown to have a positive role on seed dormancy [201]. The Arabidopsis *DOG1* gene also promotes dormancy and longevity, and its transcript abundance is positively correlated with dormancy and longevity [173,174]. Interestingly, *DOG1* has been described to strengthen the seed coat through the modulation of GA metabolism genes [204]. These results suggest the existence of an intricate relationship between GA-signalling, dormancy, and longevity.

Lastly, another study with Arabidopsis mutants defective on gibberellins synthesis and signalling (GA-deficient *ga1-*3 and GA-insensitive *gai*) reports that the role of GA in longevity appeared to be inconclusive [154]. Therefore, the relationship between GA signalling and seed longevity needs more investigation. The effects on seed longevity of genes associated with GAs studied in different plant species are summarized in Table 1 and Figure 2C.

**Table 1:** Phytohormone-related genes and their effects on seed longevity.

Gene	Longevity Regulator	Related Phytohor-Mone	Species	Ageing Conditions of Study *	Reference
*ABI3*	Positive	ABA	*Arabidopsis thaliana*	Wet	Sugliani et al. [119]; Clerkx et al. [154]
	Positive		*Solanum lycopersicum*	Dry	Livne et al. [201]
*ABI4*	Positive	ABA	*Medicago truncatula*	Wet, Dry, and Intermediate	Zinsmeister et al. [170]
*ABI5*	Positive	ABA	*Medicago truncatula*	Dry and Intermediate	Zinsmeister et al. [171]
*LEC1*	Positive	ABA	*Arabidopsis thaliana*	Wet	Sugliani et al. [119]
*FUS3*	Positive	ABA	*Solanum lycopersicum*	Dry	Livne et al. [201]
*ABA1*	Positive	ABA	*Arabidopsis thaliana*	Wet	Clerkx et al. [154]
*HSFA9*	Positive	ABA/IAA	*Arabidopsis thaliana*	Wet	Zinsmeister et al. [99]
	No effect			Intermediate	
	Positive		*Medicago truncatula*	Wet	
	No effect			Intermediate	
	Positive		*Helianthus annuus*	Dry	Carranco et al. [156]
*DREB2B*	Negative	ABA/IAA	*Arabidopsis thaliana*	Wet	Ali et al. [178]
	Negative		*Gossypium hirsutum*	Wet	Ali et al. [178]
	Positive		*Helianthus annuus*	Dry	Carranco et al. [156]
	Positive		*Zea mays*	Wet	Han et al. [190]
*NYC1*	Positive	ABA	*Arabidopsis thaliana*	Dry	Nakajima et al. [166]
*TIP3*	Positive	ABA	*Arabidopsis thaliana*	Wet	Mao and Sun [152]
*RSL1*	Positive	ABA	*Arabidopsis thaliana*	Wet and Dry	Bueso et al. [176,177]
*WHY*	Positive	ABA	*Arabidopsis thaliana*	Wet	Taylor et al. [182]; Isemer et al. [183]
*IAA27*	Negative	IAA	*Helianthus annuus*	Dry	Carranco et al. [156]
*GH3-2*	Negative	IAA	*Oryza sativa* (rice)	Wet and Dry	Yuan et al. [189]
*ATHB25*	Positive	GA	*Arabidopsis thaliana*	Wet	Bueso et al. [98]
*ATHB22*	Positive	GA	*Arabidopsis thaliana*	Wet	Bueso et al. [98]
*ATHB31*	Positive	GA	*Arabidopsis thaliana*	Wet	Bueso et al. [98]
*COG1*	Positive	GA	*Arabidopsis thaliana*	Wet	Bueso et al. [203]
*CDF4*	Positive	GA	*Arabidopsis thaliana*	Wet	Bueso et al. [203]
*LE25*	Positive	GA	*Solanum lycopersicum*	Dry	Livne et al. [201]
*GOLS1*	Positive	GA	*Solanum lycopersicum*	Dry	Livne et al. [201]
*DOG1* *SlDOG1-2*	Positive	ABA/GA	*Arabidopsis thaliana*	Dry	Benksin et al. [173]
			*Solanum lycopersicum*	Intermediate	Bizouerne et al. [148]
*PROCERA*	Positive	GA	*Solanum lycopersicum*	Intermediate	Bizouerne et al. [148]

* Ageing conditions of study: wet ageing (wet; >80% RH); intermediate ageing (intermediate; 75% RH; 30–35 °C); and dry ageing (dry; <70% RH; 25 to −20 °C).

### 5.4. Brassinosteroids and Other Hormones

Brassinosteroids (BRs) are ubiquitously distributed across different plant organs and tissues, with significantly higher concentrations observed in seeds, pollen, and fruits [205]. These compounds play a multifaceted role in plant biology, impacting various aspects of growth and development, such as seed morphology and germination, and stress responses [206,207,208]. Recent investigations have unveiled their potential negative regulation of seed longevity through a technique known as priming. Priming involves controlled seed imbibition and subsequent drying treatments typically used to improve germination performance. However, the treatment sometimes reduces seed longevity, a side effect attributed to the increase in seed coat permeability. However, priming of BR-deficient mutants (*cyp85a1/a2* and *det2*) resulted in significantly extended seed longevity [209,210]. This longevity extension in mutant seed was attributed, at least in part, to a reduction of seed coat permeability after priming, in agreement with the known positive effect of BR-signalling regulation on this process. Since BRs are hormones that regulate common physiological responses to those regulated by GAs [211,212], it would be interesting to study their possible antagonistic regulation on seed coat development.

Apart from BRs, there is little documented evidence on the role of other hormones in seed longevity. It is worth noting that mutant seeds that are ethylene-resistant1 (*etr1*) and jasmonic acid-resistant1 (*jar1-1*) have exhibited no significant loss in viability even after prolonged dry storage periods of up to four years [154], suggesting that these hormones, even being involved in seed germination [213], may not have a relevant role in ageing. Despite the growing body of research in recent years, the dearth of knowledge in the area of seed longevity and its regulation by hormones still presents a substantial scope for exploration and discovery.

## 6. Conclusions

Research on seed longevity has provided important components with roles in several cases conserved across species. However, the intricate interplay between genetics and environmental factors underlying the multifaceted nature of seed longevity requires a profound analysis of the connections between different gene regulatory networks. In addition, since seed ageing is particularly prone to variations depending on experimental set ups, it is advisable to test different storage conditions for a robust assessment of the regulatory breadth of the identified gene. Recent studies have shed light on the role of hormone signalling in the acquisition of longevity and ageing mechanisms of seeds, with a particular focus on the ABA signalling pathway, which has emerged as a significant positive regulator of seed longevity. An epistatic relationship between auxin and ABA in promoting seed longevity has been found, although it is important to consider that IAA can act as a negative regulator when interconnected pathways simultaneously reduce raffinose synthesis. Notably, despite the typical antagonistic relationship between GAs and ABA, various species have exhibited positive effects of GAs on seed longevity. Also, while GAs and BRs have been implicated in seed coat development, potentially influencing seed longevity, further research is needed to provide a comprehensive understanding of their roles. Future research directions in seed longevity will benefit by considering a global and integrated perspective on the various aspects exposed in this review, such as experimental design, species-specific traits related to seed structure and composition, and hormone metabolism and signalling.

## Data Availability

Not applicable.
